# Overexpression of *Escherichia coli udk* mimics the absence of T7 Gp2 function and thereby abrogates successful infection by T7 phage

**DOI:** 10.1099/mic.0.064527-0

**Published:** 2013-02

**Authors:** Andrey Shadrin, Carol Sheppard, Dhruti Savalia, Konstantin Severinov, Sivaramesh Wigneshweraraj

**Affiliations:** 1 Section of Microbiology, Faculty of Medicine and MRC Centre for Molecular Bacteriology and Infection, Imperial College London, SW7 2AZ, UK; 2 Waksman Institute for Microbiology and Department of Molecular Biology and Biochemistry, Rutgers, The State University of New Jersey, Piscataway, NJ 08854, USA; 3 Gene Biology of the Russian Academy of Sciences, Moscow, Russia; 4 Molecular Genetics of the Russian Academy of Sciences, Moscow, Russia

## Abstract

Successful infection of *Escherichia coli* by bacteriophage T7 relies upon the transcription of the T7 genome by two different RNA polymerases (RNAps). The bacterial RNAp transcribes early T7 promoters, whereas middle and late T7 genes are transcribed by the T7 RNAp. Gp2, a T7-encoded transcription factor, is a 7 kDa product of an essential middle T7 gene *2*, and is a potent inhibitor of the host RNAp. The essential biological role of Gp2 is to inhibit transcription of early T7 genes that fail to terminate efficiently in order to facilitate the coordinated usage of the T7 genome by both host and phage RNAps. Overexpression of the *E. coli udk* gene, which encodes a uridine/cytidine kinase, interferes with T7 infection. We demonstrate that overexpression of *udk* antagonizes Gp2 function in *E. coli* in the absence of T7 infection and thus independently of T7-encoded factors. It seems that overexpression of *udk* reduces Gp2 stability and functionality during T7 infection, which consequently results in inadequate inhibition of host RNAp and in the accumulation of early T7 transcripts. In other words, overexpression of *udk* mimics the absence of Gp2 during T7 infection. Our study suggests that the transcriptional regulation of the T7 genome is surprisingly complex and might potentially be affected at many levels by phage- and host-encoded factors.

## Introduction

The *Escherichia coli* bacteriophage T7 relies upon the multisubunit RNA polymerase (RNAp) of its host and a single subunit RNAp encoded by its own genome for transcription of its genes. During the infection process, the host RNAp transcribes early T7 phage genes from the genetic left end of the viral genome, whereas the T7 RNAp transcribes the middle and late T7 phage genes. Whilst activities of both the host and T7 RNAp on the T7 phage genome are likely to be regulated and co-ordinated at multiple levels involving host and phage factors, it is generally accepted that the products of T7 phage early gene *0.7* and middle gene *2* are responsible for inhibition of host RNAp at later stages of infection such that host resources are shifted towards the production of viral transcripts synthesized by the T7 RNAp. T7 Gp0.7 is a serine/threonine kinase that phosphorylates a specific threonine residue (T1068) in the evolutionarily variable region (β′ GNCD/I6 domain) of the host RNAp, which results in increased ρ-independent termination during transcription of host genes (Severinova & Severinov, 2006). T7 Gp2 is a 7 kDa protein that binds to the structurally conserved β′ jaw domain of the host RNAp that is adjacent to the β′ GNCD/I6 domain. Gp2 is a potent inhibitor of the *E. coli* RNAp and antagonizes conformational changes in the RNAp and interactions between the RNAp and the promoter DNA that are necessary for the engagement of the RNAp with the promoter DNA for transcription initiation (James *et al.*, 2012; Mekler *et al.*, 2011).

Unlike Gp0.7, Gp2 is essential for T7 phage growth in *E. coli*. Recently, it was demonstrated that the essential function of Gp2 during T7 phage infection is to abolish transcription from the strong host RNAp-dependent early T7 phage promoters A0–A3 (Savalia *et al.*, 2010). Down-mutations in the T7 A3 promoter compensate for the absence of Gp2 (Savalia *et al.*, 2010). Likewise, mutations in *boxA* anti-termination element located downstream of early T7 phage promoters also make Gp2 dispensable (Savalia *et al.*, 2010). It therefore follows that, in the absence of Gp2, T7 A0–A3 promoter-initiated and *boxA* anti-terminated transcription by host RNAp interferes with T7 phage infection. The exact mechanism of this interference remains unknown.

Qimron *et al.* (2006) showed that mutant T7Δ*0.7* phage, and to a relatively lesser extent wild-type T7 phage, is unable to grow on wild-type *E. coli* cells overexpressing *udk*, a uridine/cytidine kinase, which converts uridine and cytidine into the ribonucleoside monophosphates UMP and CMP, respectively. More recently, Qimron *et al.* (2008) identified missense mutations in gene *2* and gene *3.5* (which encodes T7 lysozyme – an inhibitor of the T7 RNAp) that enable T7Δ*0.7* mutant and wild-type T7 phage growth on *E. coli* overexpressing *udk*. Overproduction of Gp2 was sufficient to suppress the growth defect of T7Δ*0.7* mutant and wild-type T7 phage in *udk* overexpressing *E. coli* cells (Qimron *et al.*, 2008). Thus, it appears that during T7 phage infection of *udk* overexpressing *E. coli* cells, Gp2 is not able to inhibit the host RNAp adequately (Qimron *et al.*, 2008). However, whether *udk* overexpression directly affects Gp2 function or is mediated via an unidentified T7 phage and/or host factor(s) during T7 phage infection remains unknown. In addition, the consequence of *udk* overexpression on the biological role of Gp2 during T7 phage infection remains uncharacterized. Here, we present results from experiments in which we have addressed these issues.

## Methods

### 

#### Plasmids.

Plasmid pAS2:Gp2 and pAS2:CR44b_13 was constructed by digesting pSW33:Gp2 (Cámara *et al.*, 2010) and pAS33:CR44b_13 (Shadrin *et al.*, 2012) with *Xba*I and *Hin*dIII restriction endonucleases and the resulting 0.3 kb fragment containing T7 gene *2* and CR44b_13 was cloned into the same sites in pBAD33 (ATCC). pAS3 was constructed from pQE30 (Qiagen) by digesting with *Bsm*I and further circularization of the largest fragment. The resulting pAS3 plasmid is distinct from pQE30 only by 407 bp (*Bsm*I–*Bsm*I deletion) in the coding region of the chloramphenicol acetyltransferase gene. To make pAS3:Udk, *E. coli udk* gene was amplified by PCR using primers 5′-TTT*GGATCC*ATGACTGATCAGTCTCACCAGTG-3′ and 5′- ATC*AAGCTT*ATTCAAAGAACTGACTTATTTTCGCT-3′ (italic underlined type indicates restriction enzyme sites), digested with *Bam*HI and *Hin*dIII and cloned into the same sites of pAS3 vector.

#### Bacterial growth attenuation assays.

**Fig. 1.**
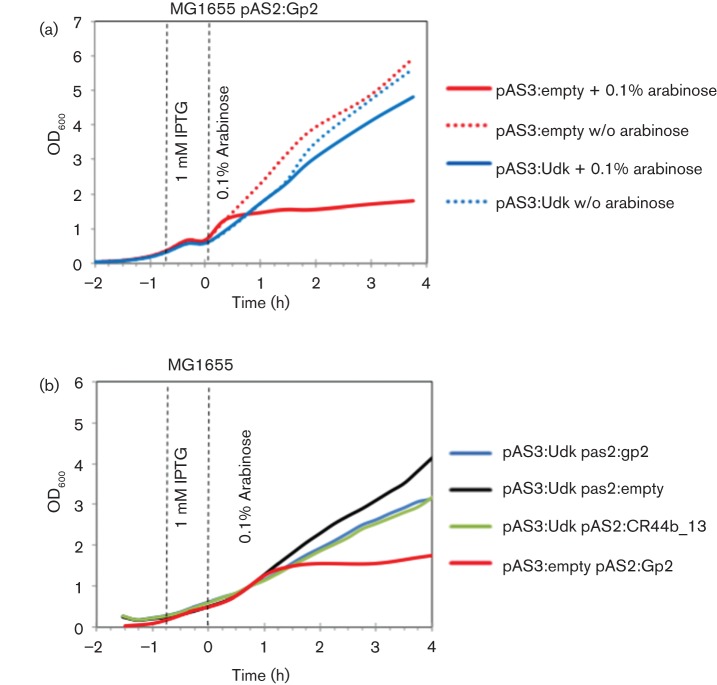
Overexpression of *udk* antagonizes Gp2 activity in *E. coli* in the absence of T7 phage infection. (a) Growth curves of *E. coli* MG1655 containing different plasmid combinations and growth conditions (as indicated). (b) As in (a), but with *E. coli* cells containing pAS2:CR44b_13 encoding Gp2-like protein CR44b_13 from CR44b phage.

**Fig. 2.**
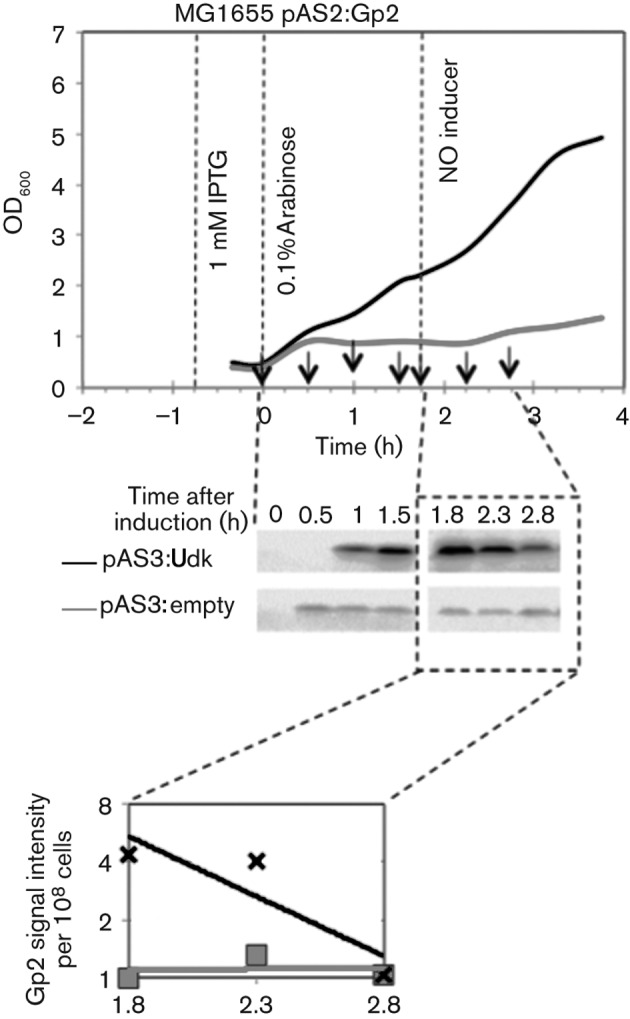
Overexpression of *udk* in *E. coli* reduces Gp2 stability and functionality in the absence of T7 phage infection. As in Fig. 1, growth curves (top panel) of *E. coli* MG1655 containing pAS2:Gp2 and pAS3:empty (grey line) and pAS2:Gp2 and pAS3:Udk (black line). Samples for preparing whole-cell extracts were taken at the indicated time points and analysed by Western blotting (middle panel), and signal intensity (relative to Gp2 signal at *t* = 1.8 in whole-cell extracts from cells containing pAS2:Gp2 and pAS3:empty) on the Western blot was quantified and plotted on a graph (bottom panel).

These assays were conducted in 250 ml flasks in a water bath at 37 °C with agitation at 180 r.p.m. Each flask contained 25 ml Luria broth (LB) growth medium supplemented with 30 mg chloramphenicol l^−1^ (to select for plasmids pAS2:Gp2 and pAS2:CR44b_13) and 100 mg ampicillin l^−1^ (to select for plasmids pAS3 and pAS3:Udk), which was inoculated with 0.25 ml overnight culture grown in the same medium additionally supplemented with 0.5 % glucose to prevent leaky expression of proteins. The overexpression of *udk* was induced by adding IPTG to 1 mM final concentration when the culture reached OD_600_~0.15. Thirty-five minutes after induction with IPTG, the cells were pelleted and resuspended in the same volume of the medium containing antibiotics. At this point a sample corresponding to *t* = 0 was taken and Gp2 synthesis was induced by adding l^+^-arabinose solution to a final concentration of 0.1 % (w/v). For the calculation of Gp2 stability, a second washing step (as above) was performed at *t* = 1.5 h to remove arabinose. At least three biological replicates were performed for each growth curve shown in Figs 1, 2 and S1, available with the online version of this paper.

#### Western blotting.

Western blots were conducted exactly as described by Shadrin *et al.* (2012) but using polyclonal antibodies against Gp2 (Eurogentec) and using anti-rabbit–horseradish peroxidase-conjugated antibodies (Dakocytomation) as secondary antibodies. Western blots were conducted for two samples obtained from two of the three biological replicates from the growth curve experiments (see above), and the best representative blot is shown. The Gp2 half-life was calculated by measuring the relative intensities of the Gp2 signal from the Western blots assuming that the Gp2 degradation begins immediately after removing arabinose.

#### Primer extension analysis of early T7 transcripts.

The reactions were conducted essentially as described by Savalia *et al.* (2010) but in *E. coli* strain BL21 containing the *udk* overexpression ASKA plasmid pAS3:Udk (Kitagawa *et al.*, 2005) in the presence of 0.5 mM IPTG.

## Results

### Overexpression of *udk* antagonizes Gp2 activity in *E. coli* in the absence of T7 phage infection

We recently described a simple bacterial growth attenuation assay to study the activity of wild-type and mutant Gp2 forms *in vivo* in the absence of a T7 phage infection (Shadrin *et al.*, 2012). In this assay, plasmid-borne Gp2 is expressed from the arabinose-inducible *araBAD* promoter in *E. coli* strain MG1655. The induction of Gp2 expression with arabinose results in efficient attenuation of bacterial growth. We adapted this assay to find out if overexpression of *udk* can antagonize Gp2 activity in the absence of a T7 phage infection. In our experimental setup, *E. coli* strain MG1655 contained two compatible plasmids: pAS2:Gp2 in which gene *2* is placed under the control of the arabinose-inducible *araBAD* promoter and either pAS3:empty or pAS3:Udk in which *udk* is placed under the control of the IPTG-inducible T5 promoter. The *E. coli* cells were grown for 1 h in LB at 37 °C to OD_600_~0.15 and *udk* overexpression was induced with 1 mM IPTG for 45 min. The cells were then washed with fresh LB media and resuspended in LB containing 0.1 % w/v l^+^-arabinose to induce Gp2 expression and allowed to grow at 37 °C. As shown in Fig. 1(a), the expected attenuation of bacterial growth upon induction of Gp2 in the absence of *udk* overexpression was detected under our experimental conditions (compare solid and dotted red lines). Overexpression of *udk*, in the absence of Gp2 expression, did not lead to attenuation of bacterial growth (Fig. 1a, dotted blue line). However, we failed to detect the expected attenuation of bacterial growth upon Gp2 induction in cells overexpressing *udk* (Fig. 1a, solid blue line). Similar results were obtained when experiments were repeated in the context of the Gp2-like protein (CR44b_13) from *Citrobacter rodentium* infective phage CR44b which shares 47 % amino acid sequence identity with Gp2 that also binds to the β′ jaw domain of the *E. coli* RNAp and inhibits transcription initiation (Fig. 1b) (Shadrin *et al.*, 2012). Overall, we conclude that the overexpression of *udk* antagonizes Gp2 function in *E. coli* in the absence of any other T7 phage encoded factor(s). In other words, it seems that the antagonistic effect of *udk* overexpression on Gp2 activity in *E. coli* during T7 phage infection occurs independently of any other T7 phage-encoded factors.

### Overexpression of *udk* in *E. coli* reduces Gp2 stability and functionality in the absence of T7 phage infection

We extended the Gp2-mediated growth attenuation assay to monitor accumulation and stability of Gp2 in the absence and presence of overexpressed Udk. The experiment shown in Fig. 2 was setup exactly as in Fig. 1, but the *E. coli* cells were subjected to an additional washing step in fresh LB to remove arabinose that is used to induce Gp2 expression. Samples were taken at *t* = 0, 0.5, 1 and 1.5 h to monitor Gp2 accumulation post-induction and at *t* = 1.8, 2.3 and 2.8 h to monitor Gp2 stability after removal of arabinose (note that all time points are relative to *t* = 0 when Gp2 expression was induced). Whole-cell extract from ~10^8^ *E. coli* cells from each sample was prepared and analysed by Western blotting using polyclonal antibodies against Gp2. As shown in Fig. 2, increased (by approximately four- to fivefold) accumulation of Gp2 was observed in whole-cell extracts prepared from *udk* overexpressing *E. coli* cells when compared with whole-cell extracts prepared from *udk* non-overexpressing *E. coli* cells. This is as expected because induction of Gp2 expression will eventually lead to inhibition of transcription of gene *2* from the pAS2:Gp2 by the *E. coli* RNAp, which, in the presence of *udk* overexpression, will not occur (see above). However, the relative stability of Gp2 in whole-cell extracts prepared from *udk* overexpressing *E. coli* cells appears to be significantly reduced upon removal of the inducer (arabinose) when compared with the stability of Gp2 in whole-cell extracts prepared from *udk* non-overexpressing *E. coli* cells (Fig. 2). Under our experimental conditions, the estimated half-life of Gp2 in *udk* overexpressing *E. coli* cells is ~49 min whereas it exceeds 3 h in *udk* non-expressing *E. coli* cells. Intriguingly, the relative amount of total Gp2 molecules in whole-cell extracts from *udk* overexpressing and non-expressing cells at *t* = 2.8 (i.e. 1 h after removal of the inducer) is comparable, even though Gp2 is unable to attenuate *E. coli* cell growth under *udk* overexpressing conditions. Since the binding of Gp2 and the promoter DNA downstream of the transcription start site to the β′ jaw domain (which is necessary for transcription initiation to occur) are mutually exclusive events (Mekler *et al.*, 2011), this observation suggests that Gp2 is somehow prevented from interacting with the RNAp under *udk* overexpressing conditions. Overall, it seems that *udk* overexpression is likely to result in a condition that mimics the absence of Gp2 function in *E. coli*.

### Overexpression of *udk* mimics the absence of Gp2 function during T7 phage infection

**Fig. 3.**
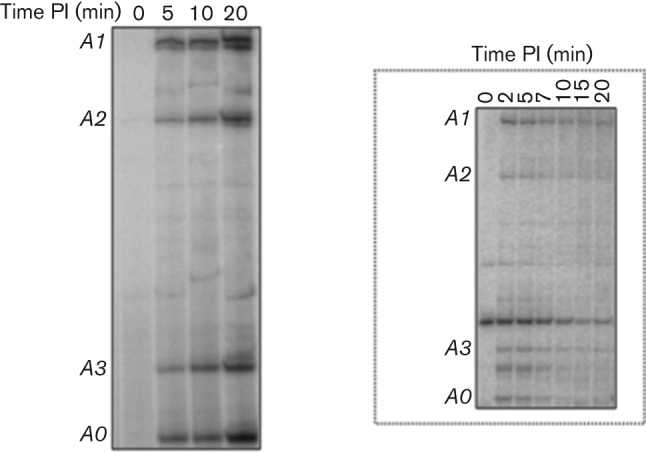
Overexpression of *E. coli udk* mimics the absence of Gp2 during T7 phage infection. Autoradiograph (8 %, w/v) showing transcripts originating from the T7 A0, A1, A2 and A3 promoters observed by primer extension analysis of RNA isolated from T7 phage-infected *E. coli* cells under *udk*-overexpressing conditions at the indicated times post-infection (PI). The inset shows the same experiment conducted with RNA isolated from T7-infected *E. coli* cells in the absence of *udk* overexpression.

The essential biological role for Gp2 during T7 infection of *E. coli* is the prevention of anti-terminated transcription from the early T7 promoters A0–A3 (Savalia *et al.*, 2010) into regions of the T7 phage genome normally transcribed by the T7 RNAp. Inspired by the finding that overexpression of *udk* could create a condition that mimics the absence of Gp2 in *E. coli*, we set out to determine the effect of *udk* overexpression on Gp2 functionality during T7 phage infection. As shown in Fig. 3 (inset) and consistent with the observation made by Savalia *et al.* (2010), in the course of wild-type T7 phage infection of *E. coli*, transcription from early T7 promoters A0–A3 by the host RNAp is eliminated shortly after initiation of transcription by the T7 RNAp (Savalia *et al.*, 2010). In contrast, when *E. coli* is infected by T7 carrying an amber mutation in gene *2*, host RNAp transcription from early phage promoters continues late in infection (Savalia *et al.*, 2010). A similar effect is observed when wild-type T7 infects a host bacterium whose RNAp lacks the β′ jaw domain (Savalia *et al.*, 2010). In agreement with the results shown in Figs 1 and 2, transcripts from early T7 promoters accumulated continuously throughout T7 phage infection in cells overexpressing *udk*. This result thus clearly indicates that overexpression of *udk* effectively prevents Gp2 function *in vivo* during T7 phage infection. In other words, overexpression of *udk* mimics the absence of Gp2 during T7 phage infection and leads to accumulation of anti-terminated transcription from early T7 promoters, which as a consequence abrogates successful T7 phage gene expression and infection.

## Discussion

In this study we demonstrate that overexpression of *E. coli udk* allows transcription from early T7 promoters to continue late in infection, thus mimicking infections that occur in the absence of Gp2 function, i.e. when *E. coli* cells are infected by gene *2* mutant T7 phage or cells whose RNAp carries lesions in the Gp2-binding β′ jaw domain is infected by the wild-type T7 phage (Savalia *et al.*, 2010). It therefore follows that *udk*, when overexpressed, obliterates the essential *in vivo* function of Gp2, i.e. inhibition of transcription from early T7 phage promoters (Savalia *et al.*, 2010). The mechanism by which overexpression of *udk* obliterates Gp2 function remains largely elusive; yet, our results indicate that it is unlikely to involve any T7 phage-encoded factors. However, we cannot exclude the possibility of the involvement of host factors that could affect Gp2 functionality during T7 phage infection under *udk* overexpressing conditions. This view is consistent with the observation that purified recombinant Udk has no detectable effect on *E. coli* RNAp transcription from the early T7 phage promoter T7 A1 on its own and does not influence the ability of Gp2 to either bind to or inhibit the *E. coli* RNAp *in vitro* (Fig. S2a, b). Further, since Udk coverts cytidine and uridine into their respective ribonucleoside monophosphates, CMP and UMP, we considered whether the significantly increased intracellular concentration of CMP and UMP under *udk* overexpressing conditions could potentially affect the ability of Gp2 to bind to and inhibit the *E. coli* RNAp. Thus, we investigated the ability of Gp2 to inhibit transcription initiation by the *E. coli* RNAp in the presence of CMP and UMP. As shown in Fig. S2(c), Gp2 is able to inhibit transcription initiation from the *lac*UV5 promoter equally well in the absence and presence of CMP and UMP. However, we note that overexpression of *udk* appears to significantly reduce the half-life of Gp2 in *E. coli* when compared with the half-life of Gp2 under *udk* non-overexpressing conditions. This could indicate that Gp2 is somehow prevented from interacting with the RNAp under *udk* overexpressing conditions. We propose that RNAp-unbound Gp2 is more susceptible to proteolysis under *udk* overexpressing conditions than Gp2 that is bound to the RNAp and thus relatively better protected from proteolysis. In support of the latter, Gp2 is relatively less stable (estimated half-life of 16 min) in mutant *E. coli* cells JE1134 (Ederth *et al.*, 2002) containing mutant RNAp without the β′ jaw domain (this is the binding site for Gp2) (Fig. S1) than in wild-type *E. coli* cells (estimated half-life of >3 h; Fig. 2). Furthermore, the view that Gp2 is somehow prevented from interacting with the RNAp under *udk* overexpressing conditions is consistent with the observation made by Qimron *et al.* (2008) demonstrating that the overexpressed *udk*-mediated antagonist effect on Gp2 function during T7 phage infection can be overcome by supplying excess plasmid-borne Gp2.

The fact that we detect continuous accumulation of transcription from early T7 phage promoters under *udk* overexpressing conditions during T7 infection is thus consistent with the view that overexpression of *udk* creates a condition that mimics the absence of Gp2 function. This provides experimental evidence in support of the model proposed by Qimron *et al.* (2008), which predicts that under *udk* overexpressing conditions, transcription by the ‘slow-elongating’ host RNAp from the early T7 promoters into regions of the T7 genome normally transcribed by the ‘fast-elongating’ T7 RNAp, causes the latter to pause, which in turn results in undue recruitment of T7 genome packaging factors Gp18 and Gp19 and thus the production of less than unit length phage genomes, which as a consequence abrogates successful infection of *E. coli* by T7 phage. We speculate that *udk* could have been part of a past mechanism in *E. coli* that existed to thwart infection by T7-like phages, but is no longer available as a mechanism for suppression of T7 infection. We propose that the T7 phage has evolved a strategy involving the serine/threonine kinase Gp0.7 to counteract the effect of Udk. Two lines of evidence support this view. Firstly, Udk is phosphorylated at a threonine residue in stationary phase *E. coli* cells (Macek *et al.*, 2008), suggesting that Udk activity could be somehow antagonized by phosphorylation. Secondly, mutant T7Δ*0.7* phage is severely impaired for growth in *E. coli* cells overexpressing *udk*, whereas wild-type T7 is relatively less impaired compared with T7Δ*0.7* phage. In other words, we propose that Gp0.7-mediated phosphorylation of Udk could prevent the Udk-mediated antagonism of Gp2 function during T7 phage infection of *E. coli*. Global transcriptome and phosphoproteome analysis in *E. coli* under *udk* overexpressing conditions and during wild-type T7 and T7Δ*0.7* infections might provide further insights into overexpressed Udk-mediated antagonism of Gp2 function in *E. coli* during T7 phage infection. Such experiments are currently under way in our laboratory.
